# Spontaneous Recanalization of Complete Internal Carotid Artery: A Clinical Reminder

**DOI:** 10.4103/2006-8808.73619

**Published:** 2010

**Authors:** Sumit Som, Bernard Schanzer

**Affiliations:** *Department of Internal Medicine and Neurology, Trinitas Regional Medical Center, Seton Hall University School of Health & Medical Sciences*

**Keywords:** Imaging, internal carotid artery, recanalization, spontaneous, surgery

## Abstract

Spontaneous recanalization of atherothrombotic extracranial cerebral arteries is rare vis-à-vis recanalization of intracranial vessels. The time course is unknown. The question is the advisability and timing of surgery in a recanalized vessel. We describe a patient with spontaneous recanalization of a totally occluded left Internal Carotid Artery (ICA) who was monitored with periodic imaging and in time had partial recanalization of the ICA. We believe patients with total ICA occlusion with return of function should be followed up with periodic carotid ultrasound, Magnetic Resonance Angiography (MRA) or CT angiography, and when appropriate, be candidates for carotid vascular interventions.

## INTRODUCTION

Spontaneous recanalization of extracranial cerebral arteries has been recognized since 1958.[1] However, it is infrequent and underreported. The intent is to underline the importance of optimum long-term monitoring of ICA occlusions and the possible benefit of surgical intervention. We describe a patient with spontaneous recanalization of a totally occluded left Internal Carotid Artery (ICA) presenting as a dense middle cerebral artery (MCA) territory infarct. The patient was monitored with periodic imaging and in time had partial recanalization of the ICA. She underwent a successful secondary preventive surgery.

## CASE REPORT

**Figure 1:**
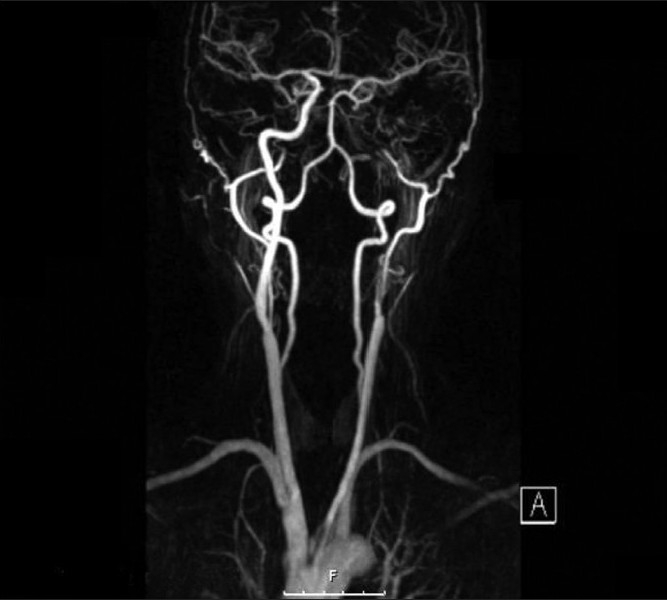
Total left ICA atherothrombotic occlusion

**Figure 2:**
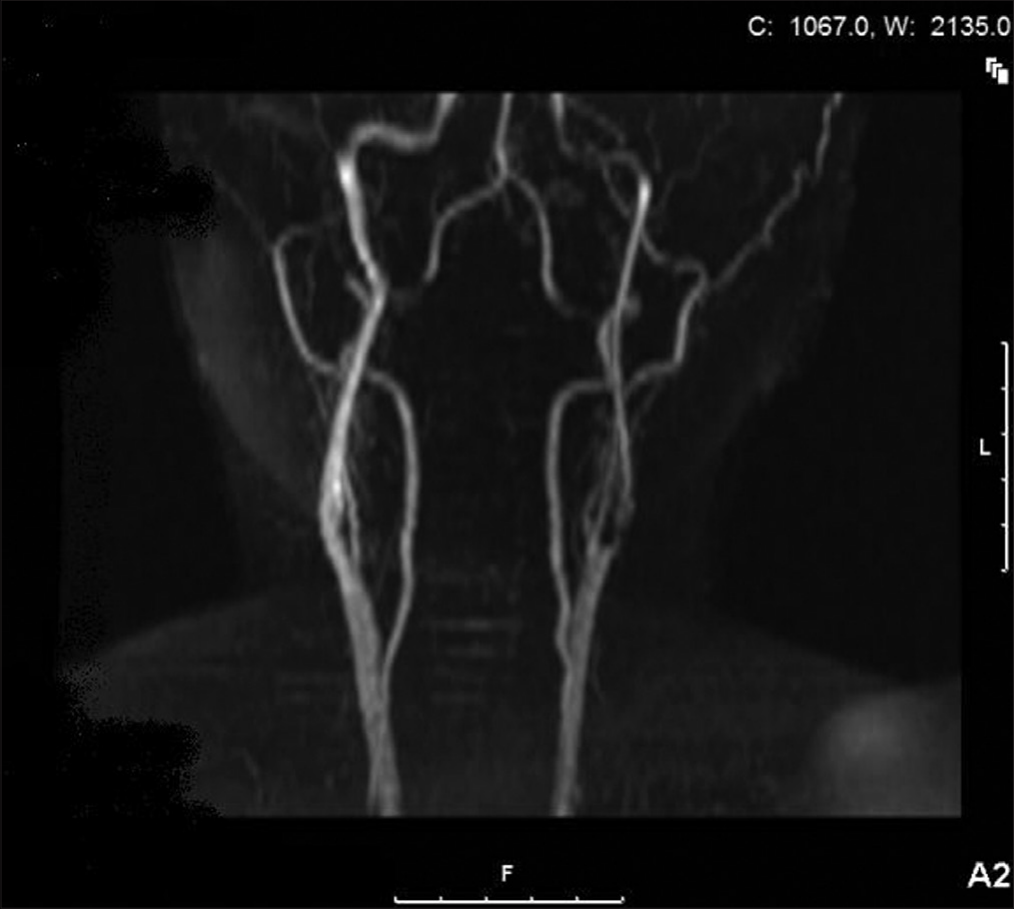
Left ICA recanalization

A 58-year-old, right-handed woman, life-long smoker, with osteoporosis, and no history of hypertension, was admitted with worsening ‘confusion’, slurring of speech, and profound right-sided weakness. Three weeks prior to admission, she had transient weakness and numbness in her right upper extremity and two days prior she had an onset of headache. Medication at home consisted of Ibandronate; she was active in her husband’s law practice. On admission, a physical examination revealed global aphasia and right hemiplegia. No carotid bruits or heart murmurs were heard. ECG, routine laboratory studies, and an initial CT scan of the head were all unremarkable. The patient was not deemed to be a candidate for TPA therapy because of late presentation, so was treated with Aspirin, Clopidogrel, and Simvastatin. The carotid duplex study revealed a complete left ICA occlusion and 41 - 59% stenosis of the right ICA. These findings were confirmed by an MRA done the day after admission, which was consistent with the total left ICA occlusion at its origin [Figure 1]. The MRI of the head showed a large infarct in the left MCA distribution. The patient stabilized and improved. Five days following the stroke she had halting dysarthric speech, with a power of 3/5 in her right extremities. Four months after the initial stroke, the patient was independent in self-care, her speech was slow, with full comprehension, and she had right spastic hemiparesis. A follow-up Carotid Ultrasound now revealed *80 - 99%* stenosis of the left ICA and unchanged 41 - 59% right ICA stenosis. This was confirmed by an MRA [Figure 2], which revealed severe proximal short segment left ICA *stenosis*, with good flow in the distal left ICA, as well as diffuse intracranial A1 segment left anterior cerebral artery disease. The patient later on had left carotid endarterectomy (CEA). Two years following the successful surgery she remains neurologically stable, is able to drive, and is with right spastic hemiparesis graded at 4/5.

## DISCUSSION

Spontaneous recanalization of the atheroembolic total ICA occlusion, although rare, does occur. The time course is unknown, probably due to varying follow-up examinations in different studies. The question is the advisability and timing of surgery in a recanalized vessel. In our case, and in similar cases, as parenchymal sparing has been found in the territory of the vessel at risk, reocclusion may occur, at which point there will be further loss of tissue and function. This patient and cohorts fall under high risk and are therefore candidates for carotid endarterectomy/stenting. Hence, patients with total ICA occlusion with return of function must be followed up with periodic carotid ultrasound, MRA or computed tomography (CT) angiography,[2] and when appropriate, become candidates for carotid vascular interventions. In addition, MRA may also be able to prioritize the subset of patients who might benefit from early (<2 weeks) carotid endarterectomy (CEA).[3] The European Carotid Surgery Trial (ECST) and the North American Symptomatic Carotid Endarterectomy Trial (NASCET),[4,5] have not only proved the benefits of CEA over the ‘best medical therapy,’ but they have also identified high-risk predictive factors for delayed stroke in those treated medically (90 to 94% stenosis, coexistent intracranial disease, contralateral occlusion, etc.). In our patient the conventional catheter angiography of the cerebral arteries has not been performed, although this could sometimes reveal the ‘string sign,’ and the diagnosis essentially depends on the MRA, which is not as sensitive and specific for detecting recanalization; nevertheless it is worth pursuing in modern clinical practice.

A number of factors affect the rate of recanalization including site of the occlusion in the arterial tree, collateral blood supply, as well as, clot-size, composition, and source. Larger clots are generally more resistant to recanalization, which might also explain why extracranial occlusions are much less likely to recanalize, compared to those in smaller intracranial vessels, as the proximal (extracranial) clots are generally bigger. Again the atherothrombotic clots are much more resistant to recanalization and lysis compared to the fibrin-rich embolic occlusion. The age of the thromoembolic material also influences the potential for recanalization, with the older ones being more resistant. Finally, a high hematocrit (increased blood viscosity causing less cerebral blood flow) is at least theoretically associated with reduced recanalization and reperfusion.
